# Monitoring nearshore ecosystem health using Pacific razor clams (Siliqua patula) as an indicator species

**DOI:** 10.7717/peerj.8761

**Published:** 2020-03-05

**Authors:** Lizabeth Bowen, Katrina L. Counihan, Brenda Ballachey, Heather Coletti, Tuula Hollmen, Benjamin Pister, Tammy L. Wilson

**Affiliations:** 1 Western Ecological Research Center, U.S. Geological Survey, Davis, CA, USA; 2 Alaska SeaLife Center, Seward, AK, USA; 3 Alaska Science Center, U.S. Geological Survey, Anchorage, AK, USA; 4 Inventory & Monitoring Program, Southwest Alaska Network, National Park Service, Fairbanks, AK, USA; 5 College of Fisheries and Ocean Sciences, Alaska SeaLife Center and University of Alaska Fairbanks, Seward, AK, USA; 6 Ocean Alaska Science and Learning Center, National Park Service, Seward, AK, USA; 7 Department of Natural Resource Management, South Dakota State University, Brookings, SD, USA

**Keywords:** Razor clam (Siliqua patula), Biomarker, Gene expression, Ecosystem health

## Abstract

An emerging approach to ecosystem monitoring involves the use of physiological biomarker analyses in combination with gene transcription assays. For the first time, we employed these tools to evaluate the Pacific razor clam (*Siliqua patula*), which is important both economically and ecologically, as a bioindicator species in the northeast Pacific. Our objectives were to (1) develop biomarker and gene transcription assays with which to monitor the health of the Pacific razor clam, (2) acquire baseline biomarker and gene transcription reference ranges for razor clams, (3) assess the relationship between physiological and gene transcription assays and (4) determine if site-level differences were present. Pacific razor clams were collected in July 2015 and 2016 at three sites within each of two national parks in southcentral Alaska. In addition to determining reference ranges, we found differences in biomarker assay and gene transcription results between parks and sites which indicate variation in both large-scale and local environmental conditions. Our intent is to employ these methods to evaluate Pacific razor clams as a bioindicator of nearshore ecosystem health. Links between the results of the biomarker and gene transcription assays were observed that support the applicability of both assays in ecosystem monitoring. However, we recognize the need for controlled studies to examine the range of responses in physiology and gene transcripts to different stressors.

## Introduction

Nearshore marine habitats worldwide are facing unprecedented challenges due to expanded commercial and industrial development, resource extraction and environmental changes, which all have the potential to significantly degrade coastal ecosystems and biological resources. Although nearshore areas in Alaska may have had fewer direct effects due to their remote nature and inaccessibility, they nevertheless are subject to impacts associated with a warming climate and potential effects of oil and gas development, tourism and harvest of marine resources.

Alterations associated with climate change, including increased temperatures, have already been noted in Alaska marine waters (Di Lorenzo & Mantua, 2016), and continuing alterations, such as acidification (Qi et al., 2017), are anticipated. Commercial and industrial developments in the Alaska nearshore, including oil and gas extraction, are increasing, with the potential to contribute contaminants to the environment that can damage coastal resources. A primary lesson from the 1989 *Exxon Valdez* oil spill is that low levels of hydrocarbons persisting in the environment can have a long-lasting effect on survival of exposed species (Ballachey et al., 2014; Esler et al., 2017).

Over the past decades, much effort has been put toward monitoring the health and productivity of nearshore ecosystems, including evaluating the composition of intertidal communities, with focused studies targeting species of economic and ecologic importance. Benthic invertebrates often are used as indicator species of ecosystem health as they are relatively sedentary, accessible and bioaccumulate contaminants and pathogens (Dean & Bodkin, 2006; Dean, Bodkin & Coletti, 2014; Livingstone et al., 2000; Bolognesi & Cirillo, 2014; Sforzini et al., 2018). However, given the complexity of nearshore systems, single species/single method analyses may provide limited information, whereas a combination of conventional and emerging tools will enhance assessment (Sforzini et al., 2018). Mussels (*Mytilus* spp., Linnaeus, 1758) have been a widely used indicator species (Beyer et al., 2017), but there are other bivalve candidates, including the razor clam. The use of razor clams as sentinels has been evaluated in China, using the Chinese razor clam, *Sinonovacula constricta* (Lamarck, 1818), an important aquaculture species (Peng et al., 2017). The Pacific razor clam, *Siliqua patula* (Dixon, 1789), may have merit as a bioindicator species in the northeast Pacific as it is important ecologically and economically (Bishop & Powers, 2003; Saeedi & Costello, 2019). Inclusion of razor clams as an indicator species, in addition to more commonly used species including mussels, may aid resource managers and enhance monitoring programs.

Razor clams are infaunal bivalves that inhabit sand substrates which are infiltrated by interstitial sea water. This habitat buffers them from the dramatic environmental fluctuations experienced by epifaunal bivalves such as mussels, which settle on a variety of surfaces from bedrock to gravel and are exposed to highs and lows of air temperature, sun exposure and wave exposure (Miller & Dowd, 2019). There are three species of razor clams found in Alaskan waters: (1) the Pacific razor clam, the largest, most widely distributed and commercially important, and the focus of this study, (2) the slightly smaller Alaska razor clam, *Siliqua alta* (Broderip & Sowerby, 1829), which occurs from Lower Cook Inlet west to the Bering Sea and Siberia and (3) a smaller subtidal species, *Siliqua sloati* (Hertlein, 1961; Foster, 1991). Pacific razor clams are harvested commercially and for personal use (subsistence and sport) and are a food source for sea otters, bears and other marine animals (Bishop & Powers, 2003; Smith & Partridge, 2004). From the 1950s to 1963, Alaska harvested the most Pacific razor clams of any state in the U.S. (National Marine Fisheries Service, 2016).

Pacific razor clams can be found in both intertidal and subtidal areas (to about 55 m depth), from the eastern Aleutian Islands, Alaska, to central California. Populations have been affected by overexploitation (Bishop & Powers, 2003; Szarzi et al., 2010), poor spawning (ADF&G, 2014) and earthquakes (Baxter, 1971). Historically, in Alaska, Pacific razor clams could be found from Southeast Alaska west across the Gulf of Alaska to the east end of the Aleutian chain (Kaiser & Konigsberg, 1977). Now they are found primarily in only a few areas, including the Alaska Peninsula, eastern Prince William Sound and the east and west coasts of Cook Inlet (ADF&G, 2010) and the status of subpopulations varies. For example, even within the relatively small area of Cook Inlet, the personal use fishery harvest of razor clams has been closed on the east side since 2015 due to low abundance estimates (ADF&G, 2018), while in contrast, the west side supports the only active commercial harvest of razor clams in Alaska (350,000–400,000 lbs/year) (ADF&G, 2019, personal communication) and a personal use fishery with no harvest limit (ADF&G, 2019).

Traditional bivalve health assessments used in environmental monitoring include biomarker assays that provide information on how an organism is functioning (Dahlhoff, 2004). A suite of biomarker assays can be used to evaluate various aspects of physiology to detect the effects of environmental and anthropogenic stressors on overall health of the bivalve. Changes in tissue and shell growth due to factors such as increased temperature, pollution, acidification or eutrophication can be detected by measuring the condition factor and shell thickness of the bivalve (Carmichael, Shriver & Valiela, 2004; Gagné et al., 2007; Gaylord et al., 2011). Hemocytes are the primary cell in the bivalve immune system and they phagocytose antigens and release reactive oxygen species as a killing mechanism (Pipe, 1990). Suppression of the immune response by contaminants can lead to disease susceptibility and pathogen exposure can stimulate an immune response (Akaishi et al., 2007; Gagné et al., 2002; Pipe & Coles, 1995). The immune status of bivalves can be evaluated by determining the number of circulating hemocytes and measuring hydrogen peroxide production. The RNA:DNA ratio assesses the metabolic condition of bivalves, because DNA levels remain constant in the cell while RNA varies based on intrinsic status and/or extrinsic perturbations. Therefore, short-term growth or stress can be identified by fluctuations in the RNA:DNA ratio (Caldarone et al., 2001; Lesser et al., 2010). The cytochrome P450 enzyme catalyzes reactions involving xenobiotics, and increased activity can reveal the presence of a pollutant (Rewitz et al., 2006). Changes in heat shock protein levels suggest exposure to a stressor, such as increased temperature or a contaminant (Cruz-Rodriguez & Chu, 2002).

Transcriptomics, the molecular investigation of changes in gene transcription, offers a new approach for assessing mechanisms by which stressors may affect organisms. The earliest observable signs of health impairment can be altered levels of gene transcripts, evident prior to clinical manifestation (Farr & Dunn, 1999; McLoughlin et al., 2006; Poynton & Vulpe, 2009). Gene transcription is the process by which information from the DNA template of a particular gene is transcribed into messenger RNA (mRNA) and eventually translated into a functional protein. The amount of mRNA transcribed from a particular gene is physiologically dictated by a number of intrinsic and extrinsic factors and analysis of mRNA can provide information about changes in the physiological state of an organism. Alterations (including increased or decreased amounts of mRNA) of transcribed genes indicative of physiological processes at the cellular level can identify exposure to stressors and elucidate mechanisms by which these stressors may have deleterious effects (Snell, Brogdon & Morgan, 2003; McLoughlin et al., 2006; Mos et al., 2008; Wu et al., 2008; Poynton & Vulpe, 2009; Miller et al., 2011; Evans & Hofmann, 2012).

Relatively little is known about the physiological or molecular responses of razor clams to environmental stimuli (Niu et al., 2013), as research into these processes has been minimal. Biomarker and gene-based diagnostics provide an opportunity to assess the health of individuals and populations of razor clams. However, a key requirement for the application of diagnostic technology in monitoring programs is the establishment of normal or “reference” ranges of values, to facilitate differentiation of natural variation among individuals from changes associated with altered environmental influences. Reference ranges can be determined via controlled laboratory experiments, where individual stressors are presented to organisms in systems devoid of multiple variables. Due to the absence of background noise, these studies should provide clearly defined reference ranges. However, interpretation of these ranges when applied to natural systems with multiple fluctuating variables can be problematic. Alternatively, reference ranges can be developed in natural systems, using location and point in time as references for comparisons with future monitoring efforts (i.e., longitudinal sampling; McLoughlin et al., 2006).

In this study, we hypothesized that biomarker and gene transcription assays on Pacific razor clams would provide complementary results, with no significant differences detected among sites considered to be relatively pristine. The sites were expected to provide a range of representative natural conditions to generate reference baseline data applicable to Pacific razor clams in this region.

Our specific objectives were to (1) develop biomarker and gene transcription assays with which to monitor the health of the Pacific razor clam, (2) acquire baseline biomarker and gene transcription reference ranges for Pacific razor clams, (3) assess the relationship between physiological and gene transcription assays and (4) determine if site-level differences were present.

## Materials and Methods

This study was conducted concurrently with a similar study on mussels, recently reported by Counihan et al. (2019). Study areas (Lake Clark National Park and Preserve, LACL and Katmai National Park and Preserve, KATM) were the same although the specific collection substrates within each park varied for mussels (protected mixed-sediment) vs. razor clams (exposed soft sediment). As analytical methods for the two studies were identical, data were collected as previously described in Counihan et al. (2019), but the specific genes targeted in the mRNA transcription varied for each species.

### Study organisms

**Figure 1:**
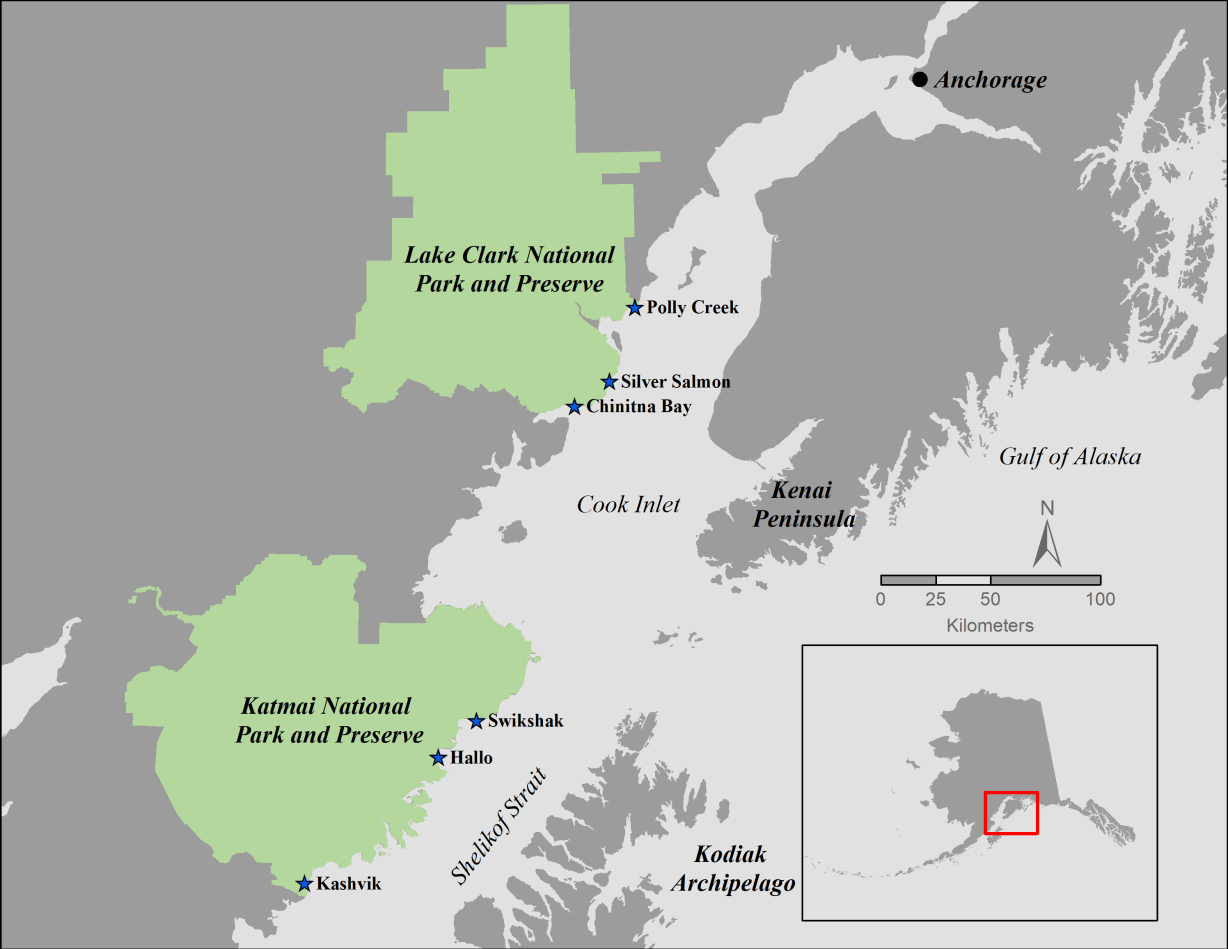
Razor clam sampling locations. Razor clams were collected in July 2015 and 2016 at three sites within each of two national parks in southcentral Alaska: Lake Clark National Park and Preserve (sites: Polly Creek, Silver Salmon and Chinitna Bay) and Katmai National Park and Preserve (sites: Hallo Bay, Swikshak and Kashvik Bay).

Pacific razor clams were collected in July 2015 and 2016 at intertidal sites within each of two national parks in southcentral Alaska: Lake Clark National Park and Preserve (sites: Polly Creek, Silver Salmon and Chinitna Bay) and Katmai National Park and Preserve (sites: Hallo Bay, Swikshak and Kashvik Bay) (Fig. 1) (National Park Service permit numbers CF-15-088 and CF-16-089). In 2015, Pacific razor clams were collected from 2 sites at KATM and 3 sites at LACL and in 2016, from 3 sites at each park. Due to logistical constraints, in 2015 we were unable to collect razor clams from Hallo Bay in KATM or take morphometrics in the field on razor clams from Chinitna Bay and Polly Creek in LACL. Approximately 20 Pacific razor clams were collected from each site, with 10 designated for biomarker assays and 10 for gene transcription (Tables S1 and S2). In LACL, beaches sampled were those known to be used for recreational clamming, ensuring presence of razor clams. In KATM, several sites were identified a priori based on historical razor clam distribution (Kaiser & Konigsberg, 1977) and initial site visits were made to determine presence of razor clams. All sites sampled had razor clams present and all razor clams were collected on the morning rising tide.

Pacific razor clams were processed as soon as possible following collection, generally within 1–3 h. For biomarker assays, hemolymph was removed by inserting a tuberculin needle into the anterior adductor muscle, aspirating the hemolymph and transferring it to a cryovial. The hemolymph and razor clam were frozen in liquid nitrogen for transport to the lab where they were transferred to a −80 °C freezer until processing. For molecular assays (gene transcription), gill tissue was removed and placed in cryovials with RNAlater^®^. The samples were kept at –20 °C until used in experiments. The length of collected razor clams ranged from 28.5 to 158.3 mm and age, as determined by counting annuli, ranged from 2 to 9 years. The maximum recorded length for *S. patula* in Alaska is 304 mm (Foster, 1991) and age is 18 years (ADF&G, 2020)

### Invertebrate biomarkers

The seven invertebrate biomarker assays detailed below were selected to assess the physiological status of the razor clams as an indicator of nearshore ecosystem health (Table 1) and have been used previously in our lab (Counihan, 2018; Counihan et al., 2019). The methods are based on published research on *Mytilus* spp.; however, these methods were validated for use with razor clams in our laboratory prior to conducting this project (K. Counihan, 2014, unpublished data).

**Table 1:** Seven invertebrate biomarker assays selected to assess razor clam and ecosystem health.

	Biomarker	Biological process	Environmental interaction
1	Condition factor	Growth	Ocean acidification Nutrient availability Contaminants Temperature
2	Shell thickness	Growth	Ocean acidification Nutrient availability Predation Density
3	Hemocyte count	Immune function	Contaminants Pathogens
4	Hydrogen peroxide	Immune function	Contaminants Pathogens
5	RNA:DNA	Metabolic condition	Nutrient availability
6	Cytochrome P450	Detoxification	Contaminants
7	Heat Shock Protein 40	Thermal stress	Stress

### Morphometrics

Frozen razor clams were thawed on ice and their length, width, height and total wet weight were measured prior to dissection. The posterior adductor muscle and digestive gland were excised for assays. The remaining soft tissue was discarded and the shell weighed. The condition factor was calculated by dividing the total razor clam weight by the shell length. Soft tissue weight ratio was calculated by dividing the soft tissue weight by the total razor clam weight (Gagné et al., 2008).

### Shell thickness

Razor clam shells were dried in a 37 °C incubator overnight and then a micrometer was used to measure the shell thickness at five regularly spaced points around the shell, approximately 1 mm from the edge (Versteegh & Hansson, 2012).

### Hemocyte count

A 0.01 ml sample of hemolymph was diluted 1:1 with tris-buffered saline (TBS) and the number of cells were counted using a hemocytometer in three replicates (Akaishi et al., 2007).

### Hydrogen peroxide production

Hemolymph samples were diluted 1:1 with TBS solution. Samples were tested in triplicate, if enough hemolymph was available, in a spectrophotometric assay by pipetting 0.05 ml of the 1:1 hemolymph:TBS solution into a 96-well plate. After incubating the plate in the dark for 1 h, 0.05 ml of phenol red solution (phosphate buffered saline pH 7.4, 5.5 mM dextrose, 0.56 mM phenol red, 8.5 U ml^−1^ horseradish peroxidase, type II) was added to each well and incubated for another 30 min in the dark. The reaction was stopped by adding 0.01 ml of 1 N NaOH, and the plate was read on a Molecular Devices SpectraMax Plus microplate reader (Sunnyvale, CA, USA) at 620 nm (Akaishi et al., 2007).

### RNA:DNA ratio

Half of the posterior adductor muscle was homogenized with a mortar and pestle and 0.15 ml of 1% sarcosyl tris-EDTA (STEB) was added to the homogenate. The mixture was vortexed for 60 min to completely dissolve the tissue. Each sample had 1.35 ml of tris-EDTA (TE) buffer added. The sample was centrifuged 15 min at 14,000×*g* at room temperature and the supernatant saved for testing. The samples were diluted 1:20 and 0.075 ml of each sample was added to a 96-well plate in duplicate. Genomic, unsheared DNA from calf thymus (Sigma–Aldrich, St. Louis, MO, USA) was used to prepare a DNA standard curve (0.1 μg ml^−1^, 0.2 μg mL^−1^, 0.4 µg ml^−1^, 0.8 µg ml^−1^, 1.6 µg ml^−1^, 3.2 µg ml^−1^, 6.4 µg ml^−1^, 10.0 µg ml^−1^). RNA from bovine pancreas (Sigma–Aldrich) was used for a RNA standard curve (0.4 µg ml^−1^, 0.8 µg ml^−1^, 1.6 µg ml^−1^, 3.0 µg ml^−1^, 6.0 µg ml^−1^, 8.0 µg ml^−1^, 12.0 µg ml^−1^ and 16.0 µg ml^−1^). The stock solutions of DNA and RNA were diluted in 0.1% STEB to the appropriate concentrations and 0.075 ml of each standard was added to the plate. The wells had 0.075 ml of ethidium bromide solution (2 µg ml^−1^) added to them and the microplate was shaken for 15 min. The plate was read in a SpectraMax Gemini EM fluorescent microplate reader (Molecular Devices) with 525 nm excitation, 600 nm emission to determine the total nucleic acid reading. Each well had 0.0075 ml of RNase solution (20 U ml^−1^) added to it and the plate was shaken for 20 min and read on the microplate reader again with the same settings. The second reading was the DNA only reading. The RNA content was determined by subtracting the second reading from the first (Caldarone et al., 2001).

### Cytochrome P450 activity

The digestive gland was homogenized in buffer (25 mM Hepes, 125 mM NaCl, 0.1 mM EDTA, 0.1 mM dithiothreitol) at a 1:5 weight:volume ratio. The mixture was centrifuged at 1,500×*g* for 10 min at 2 °C and the supernatant transferred to a clean tube. The supernatant was centrifuged at 10,000×*g* for 20 min at 2 °C. The supernatant was discarded and the pellet resuspended in 0.15 ml of microsome buffer (25 mM Hepes, 140 mM NaCl, 1 mM KH_2_PO_4_). A microsome sample was incubated with 30 uM rifampin for 48 h to be used as a positive control. A 96-well plate was inoculated with 0.05 ml of each microsome sample in triplicate, and a media only blank was included. Each well had 0.05 ml of 50 µM BFC solution (7-benzyl-4(trifluoromethyl) coumarin in phenol red-free Dulbecco’s Modified Eagle Medium) added and the plate was incubated for 4 h at room temperature. The plate had 0.04 ml of stop solution (80% CH_3_CN, 20% 0.5 M Tris base) added to each well and it was read on a SpectraMax Gemini EM fluorescent microplate reader (Molecular Devices) at 410 excitation, 530 emission (Mensah-Osman et al., 2007).

### Heat shock protein

Half of the posterior adductor muscle was homogenized and lysed in buffer (150 mM NaCl, 1% Triton-X 100, 0.5% sodium deoxycholate, 0.1% sodium dodecyl sulphate, 50 mM Tris, 1 mM phenylmethylsulfonyl fluoride). The homogenate was centrifuged at 12,000 rpm for 20 min at 4 °C and the supernatant collected. The protein concentration was determined using a Bradford assay. The sample was mixed 1:1 with 2X Laemmli buffer, boiled at 100 °C for 5 min and loaded on a SDS-PAGE gel. Each gel was run with a positive heat shock protein control and a molecular weight marker. The gel was electrophoretically transferred onto a polyvinyl difluoride (PVDF) membrane. Total protein was visualized by staining the gel with Pierce reversible protein stain (Thermo Scientific, Pittsburgh, PA USA). The stain was removed and the membrane was probed with a mouse anti-HSP40 primary antibody (Abcam, Cambridge, MA USA). The membrane was washed and incubated with a secondary alkaline phosphatase labeled anti-mouse antibody (Abcam). The membrane was washed again and alkaline phosphatase substrate added. The membrane was photographed to document bands and analyzed with Image Studio Lite software, version 5.2.5 (Li-Core, Lincoln, NE, USA).

### Invertebrate gene transcription

Five genes of interest and two reference genes were quantified using the quantitative polymerase chain reaction (qPCR) technique to assess the status of the razor clams as an indicator of nearshore ecosystem health (Table 2). The transcript panel utilized here was developed based on studies of clams subjected to single or multiple stressors.

**Table 2:** Genes selected to assess razor clam and ecosystem health.

Gene	Function
Calmodulin (CaM)	Shell formation—indication of ocean acidification; metabolism, immune response (Chen et al., 2012; Leite et al., 2013)
Ferritin (Ferr)	Increased in response to pathogens, metabolizing iron (Li et al., 2011; Perrigault, Tanguy & Allam, 2009)
Heat shock Protein 70 (HSP70)	Thermal stress; bacteria exposure (Cellura, Toubiana & Roch, 2007)
Heat shock Protein 90 (HSP90)	Responds to contaminants; temperature, salinity change, metabolism; provides cellular protection (Choi et al., 2006; Choi, Jo & Choi, 2008; Gao et al., 2007).
Peptidylprolyl isomerase A (PPIA)	Proinflammatory, increased in response to pathogen stimulus (Wang et al., 2013)
18S	Reference (Niu et al., 2014; Moreira et al., 2012)
Elongation Factor Alpha-1 (EF1a)	Reference (Araya et al., 2008; Mateo et al., 2010)

Genes were selected for the transcript panel based on their known responses to stressors, including pathogens, xenobiotics, ocean acidification, heat stress and dissolved oxygen levels (Choi et al., 2006; Choi, Jo & Choi, 2008; Cellura, Toubiana & Roch, 2007; Gao et al., 2007; Perrigault, Tanguy & Allam, 2009; Li et al., 2011; Chen et al., 2012; Niu et al., 2013; Leite et al., 2013; Wang et al., 2013).

### Tissue collection and RNA extraction

Gill tissue was collected from each razor clam and placed into RNAlater^®^ (Ambion/Life Technologies, Grand Island, NY, USA). All tissue samples were stored at −80 °C. Total RNA was extracted from pulverized gill tissue using the RNeasy Lipid Tissue Mini Kit (Qiagen; www.qiagen.com). To remove contaminating genomic (g)DNA, the spin columns were treated with 10 U μl^−1^ of RNase-free DNase I (DNase, Amersham Pharmacia Biotech Inc.; www.apbiotech.com) at 20 °C for 15 min. RNA was then stored at −80 °C pending further analyses.

### cDNA synthesis

A standard cDNA synthesis was performed on 2 μg of RNA template from each razor clam. Reaction conditions included 4 units reverse transcriptase (Omniscript, Qiagen, Valencia, CA, USA), 1 μM random hexamers, 0.5 mM each dNTP, and 10 units RNase inhibitor, in RT buffer (Qiagen, Valencia, CA, USA). Reactions were incubated for 60 min at 37 °C, followed by an enzyme inactivation step of 5 min at 93 °C and then stored at −20 °C until further analysis.

### Primer design

Degenerate primers were designed based upon multi-species alignments (GenBank). Briefly, degenerate primer pairs developed for the razor clam were used on cDNA from three randomly selected razor clam samples. Degenerate primer pairs were designed to amplify five genes of interest and two ribosomal housekeeping genes (Tables 2 and 3). The PCR amplifications using these primers were performed on 20 ng of each cDNA sample in 50 μl volumes containing 20–60 pmol of each primer, 40 mM Tris-KOH (pH 8.3), 15 mM KOAc, 3.5 mM Mg (OAc)2, 3.75 μg ml^−1^ bovine serum albumin (BSA), 0.005% Tween-20, 0.005% Nonidet-P40, 200 μM each dNTP, and 5U of Advantage^®^ 2 Taq polymerase (Clontech, Palo Alto, CA, USA). The PCR was performed on an MJ Research PTC-200 thermal cycler (MJ Research, Watertown, MA, USA) and consisted of 1 cycle at 94 °C for 3 min, and then 40 cycles at 94 °C for 30 s, at 60 °C for 30 s, and 72 °C for 2 min, with a final extension step of 72 °C for 10 min. The products of these reactions were electrophoresed on 1.5% agarose gels and resulting bands visualized by ethidium bromide staining. Definitive bands representing PCR products of a predicted base pair size of the targeted gene were excised from the gel, and extracted and purified using a commercially available nucleic acid-binding resin (QIAEX II Gel extraction kit; Qiagen, Valencia, CA, USA).

**Table 3:** *Siliqua patula*-specific quantitative real-time polymerase chain reaction primers used in the analysis of Pacific razor clams.

Gene	Forward primer	Sequence (5′ → 3′)	Reverse primer	Sequence (5′ → 3′)
*CaM*	Sipa CaM F	GATGCTGATGGTAATGGAACA	Sipa CaM R	CTYAGTTCAGCTGCACTAATA
*EF1a*	Sipa EF1a F	ATCTCTGGATGGCACGGAG	Sipa EF1a R	ATAGCATCCAAAGCCTCTAGT
*FERR*	Sipa Ferr F	ACTACTTCGACCGTGATGATAT	Sipa Ferr R	CGCGACCACCTCTCTTGTTC
*HSP70*	Sipa Hsp70 F	GAACCACACCAAGTTATGTG	Sipa Hsp70 R	CTACCAATGAGACGCTTGGC
*HSP90*	Sipa Hsp90 F	CTCCAGGCTGGAGCTGATA	Sipa Hsp90 R	ACCTCCAGCTGCAGACTC
*PPIA*	Sipa PPIA F	ACTTCCAACTGAAGCATACTG	Sipa PPIA R	TTCCATCCAACCAGCTAGTT
*18S*	My 18s F	GTGCTCTTGACTGAGTGTCTCG	My 18s R	CGAGGTCCTATTCCATTATTCC

### Real-time PCR

Real-time PCR reactions for the individual, Pacific razor clam-specific housekeeping genes (18S and EF1-a) and genes of interest were run in separate wells (Tables 2 and 3). Briefly, 1 μl of cDNA was added to a mix containing 12.5 μl of Applied Biosystems Fast SYBR Green^®^ Master Mix (5 mM Mg 2+) (Qiagen, Valencia, CA, USA), 0.5 μl each of forward and reverse sequence specific primers (Invitrogen, Carlsbad, CA, USA) and 10.5 μl of RNase-free water; total reaction mixture was 25 μl. The reaction mixture cDNA samples for each gene of interest and reference genes were loaded into Fast 96 well plates in duplicate and sealed with optical sealing tape (Applied Biosystems, Foster City, CA, USA). Reaction mixtures that contained water but no cDNA were used as negative controls.

### Statistical analysis

Both reference genes were evaluated for stability and ranked using the web-based analysis tool RefFinder (https://www.heartcure.com.au/for-researchers/) (Chen et al., 2015). Due to the differential stability of the reference genes, cycle threshold crossing values (C_T_) for the genes of interest were normalized to the most stable reference gene (18S).

Analysis of qPCR data was conducted using normalized values (housekeeping gene threshold crossing subtracted from the gene of interest threshold crossing); the lower the normalized value, the more transcripts are present. A change in normalized value of 2 is approximately equivalent to a 4-fold change in the amount of the transcript.

For all data (gene transcription and biomarker assays), medians, 2.5% and 97.5% percentiles and ranges were calculated (NCSS, Statistical and Power Analysis Software, Kaysville, UT, USA). We used Gaussian linear random effects models to estimate site means for each gene transcription factor and biomarker parameter. We included sampling year as a random effect to account for variances introduced by collecting sample units over the course of 2 years. We fit separate models for each gene with maximum likelihood estimation using the lme4 package in R 3.5.0 (R Development Core Team, 2018), which accounts for the unbalanced data using Satterthwaite’s method. We conducted post-hoc Tukey tests of site level differences. To obtain site means for each year, we fit a Gaussian linear model with a site by year interaction. The resulting site means were used to test for correlations between genes and biomarker parameters. Relationships between gene transcript and biomarker data, as well as within gene transcript and biomarker data, were assessed in *R* using Pearson correlations.

We conducted two-dimensional non-parametric multidimensional scaling of the Bray–Curtis dissimilarity from gene transcripts (*N* = 112) and biomarkers (*N* = 73) using the Vegan package in R version 3.5.0. The graphical representations show individual razor clams clustered by similarity in transcription and biomarker values and not by pre-defined groups such as location. We obtained vectors describing the strength of each gene and biomarker contribution to the two non-metric multidimensional scaling (NMDS) axes for graphical display. We evaluated goodness of fit for NMDS models using stress plots.

## Results

Over the 2 years, approximately 20 Pacific razor clams were collected from each site (Hallo Bay at KATM was not sampled in 2015 due to logistical constraints). A total of 116 razor clams were used for biomarker assays, of which 73 had a full complement of biomarker data (missing data due to logistical constraints of taking morphometric measurements in the field for razor clams from Chinitna Bay and Polly Creek in 2015) and 112 were used for gene transcription. The razor clams ranged in length from 28.5 to 158.3 mm and were generally larger at LACL (86.1–158.3 mm) than at KATM (28.2–103.7 mm). As size clearly varied, and presumably was an indication of age, we estimated age based on annuli counts. Age was tested (lme4 package, R 2.8.1; R Development Core Team, 2012) and no significant effects on razor clam biomarkers or gene transcription were noted; thus neither age nor size were considered in further analyses.

### Reference ranges and correlations

Biomarker and gene transcript medians, 2.5% and 97.5% percentiles, and ranges were created for use in future monitoring efforts (Table 4). Site-specific medians and ranges for the biomarker and gene transcription assays during each year are provided as Supplemental Material (Tables S1 and S2).

**Table 4:** Medians, 2.5% and 97.5% percentiles and ranges for all variables across all sites and years (for gene transcription, higher numbers indicate less transcription and a default value of 34 indicates a quantity less than detection limits).

**Condition factor**			**Shell thickness**			**Hemocyte count**		
Median	2.5–97.5%	Range	Median	2.5–97.5%	Range	Median	2.5–97.5%	Range
0.38	0.05–1.43	0.05–1.91	0.36	0.13–0.78	0.11–1.90	9.92	1.17–58.33	0.67–163.50
**Hydrogen peroxide**			**RNA:DNA ratio**			**P450 Activity**		
Median	2.5–97.5%	Range	Median	2.5–97.5%	Range	Median	2.5–97.5%	Range
0.05	0.02–0.11	0.01–0.12	59.66	4.91–408.25	0.63–454.10	46.14	16.73–132.95	13.11–146.37
**HSP40**			**CaM**			**Ferr**		
Median	2.5–97.5%	Range	Median	2.5–97.5%	Range	Median	2.5–97.5%	Range
1.24	0.00–7.14	0.00–17.79	8.12	6.77–9.62	6.28–10.39	1.17	−1.22–2.76	−1.56–3.78
**HSP70**			**HSP90**			**PPIA**		
Median	2.5–97.5%	Range	Median	2.5–97.5%	Range	Median	2.5–97.5%	Range
5.47	2.71–7.81	1.52–8.03	5.50	2.45–6.59	2.05–6.69	7.67	0.40–9.40	0.05–10.36

**Figure 2:**
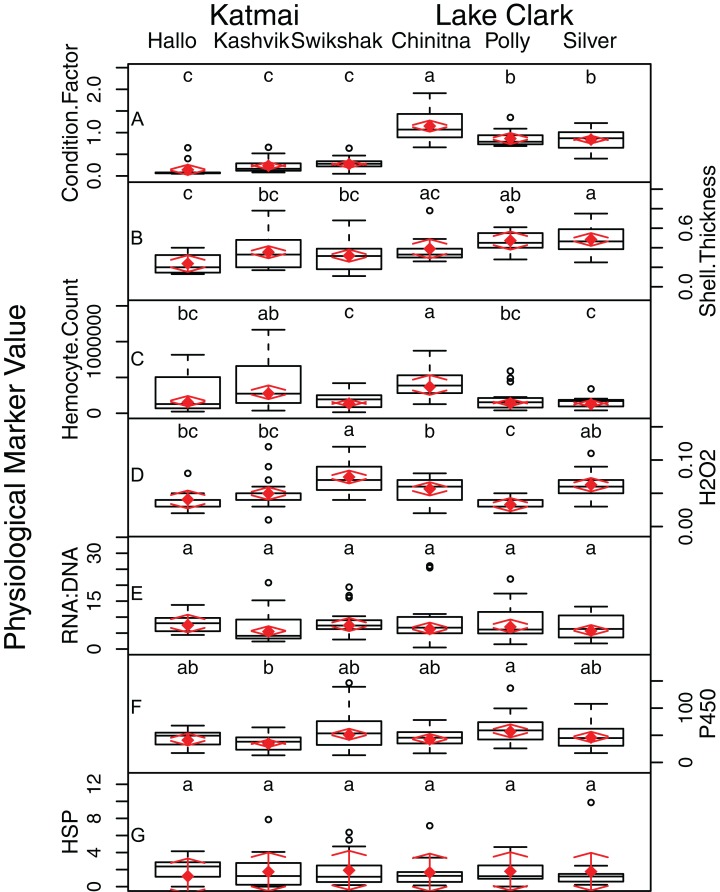
Boxplots of data from seven different physical biomarker assays (A–G) performed on Pacific razor clams collected at six sites in LACL and KATM National Parks and Preserves, Alaska, USA. Random effects model results are denoted by red diamonds (mean) and red arrows (95% confidence intervals). Sites sharing a letter did not differ statistically based on post-hoc testing (*P* < 0.05). For example, Condition Factor was significantly different between Chinitna and all other sites, while Polly and Silver were not significantly different from each other.

**Figure 3:**
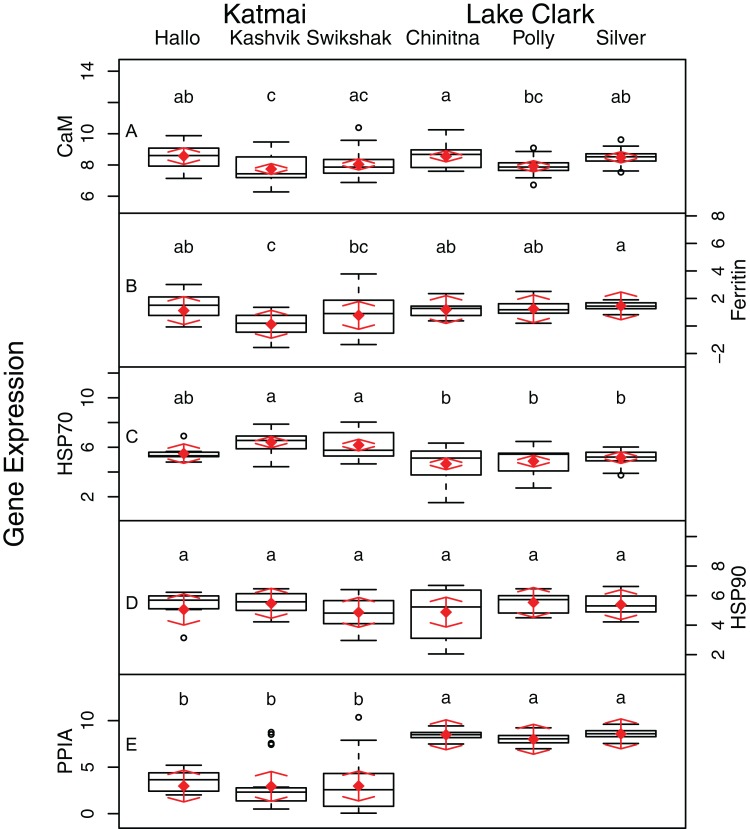
Boxplots of gene transcription data (A–E) obtained from razor clams collected at six sites in Lake Clark and Katmai National Parks and Preserves, Alaska, USA. Random effects model results are denoted by red diamonds (mean), and red arrows (95% confidence intervals). Sites sharing a letter did not differ statistically based on post-hoc Tukey testing (*P* < 0.05).

**Figure 4:**
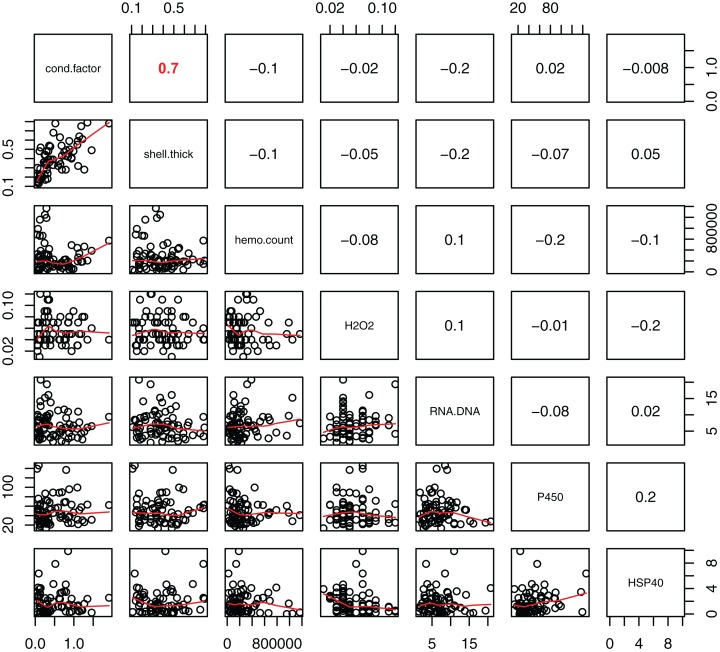
Pearson correlations between biomarkers obtained from 120 razor clams collected at six sites in Lake Clark and Katmai National Parks and Preserves, Alaska, USA. Pearson correlations between biomarkers obtained from 120 razor clams collected at six sites in LACL and KATM National Parks and Preserves, Alaska, USA. Bold red numbers are statistically significant correlations (*P* < 0.001).

**Figure 5:**
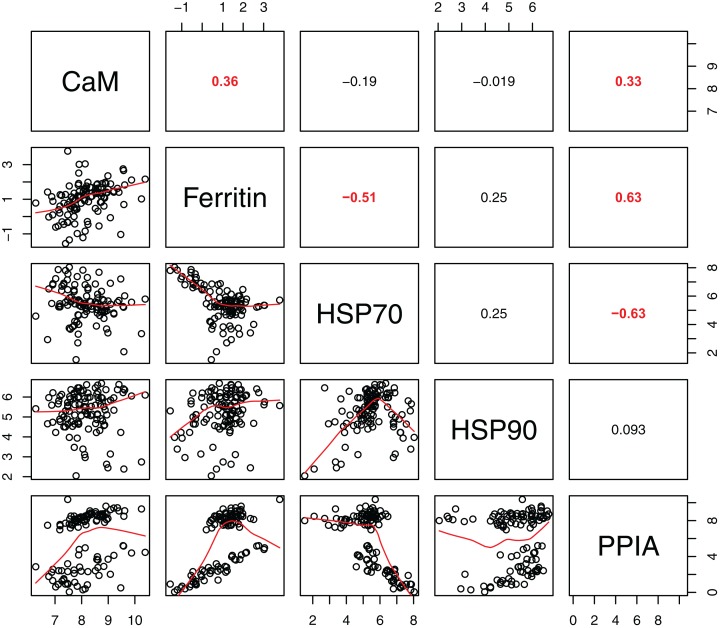
Pearson correlations between gene transcripts obtained from 120 razor clams collected at six sites in LACL and KATM National Parks and Preserves, Alaska, USA. Pearson correlations between gene transcripts obtained from 120 razor clams collected at six sites in LACL and KATM National Parks and Preserves, Alaska, USA. Red numbers are statistically significant correlations (*P* < 0.001).

Statistically significant differences among sites were identified for five of the seven biomarkers (condition factor, shell thickness, hemocyte count, H_2_O_2_ and P450; Fig. 2), and four of the five genes (CaM, Ferr, HSP70, and PPIA; Fig. 3). One statistically significant positive correlation within the biomarker assay was present between condition factor and shell thickness (Fig. 4). Within the gene transcription assays, statistically significant positive correlations were identified between: CaM and Ferr, CaM and PPIA, and Ferr and PPIA (Fig. 5). Statistically significant negative correlations within the gene transcription assays were identified between: CaM and HSP70 and PPIA and HSP70 (Fig. 5). Interestingly, both populations are segregated by PPIA transcript levels. No negative correlations within the biomarkers were identified. Significant correlations (*P* ≤ 0.05) between the biomarker and gene transcription results also were identified (Table 5). For interpretation of results, we considered correlations with *P* values that were >0.30 (or <−0.30) to be of possible biological significance. For these calculations, the mean values for each site-year were used, as biomarkers and gene transcription were not measured on the same individual razor clams (and thus the sample size was lower, at *n* = 9). Regarding the gene transcription-biomarker correlations only (Table 5), as a larger normalized value is indicative of lower transcription levels, a negative *R* value actually indicates a positive relationship, and a positive *R* value indicates a negative relationship. A positive correlation was observed between Ferr transcription and hemocyte count and negative correlations between PPIA transcription and condition factor, as well as PPIA transcription and shell thickness.

**Table 5:** Results of the Pearson correlations between gene transcripts and biomarkers obtained from Pacific razor clams collected at six sites in Lake Clark and Katmai National Parks and Preserves, Alaska, USA. Statistically significant correlations (*P* < 0.05) are bolded. Values reflect the numerical correlation; however, the biological relationship will be the inverse, as lower C_T_ values reflect higher levels of gene transcription.

		CaM	Ferr	HSP70	HSP90	PPIA
1	Condition factor	0.00	−0.017	−0.30	0.083	**0.70**
2	Shell thickness	0.00	−0.083	−0.25	0.28	**0.72**
3	Hemocyte count	−0.37	**−0.75**	0.68	0.20	−0.28
4	H_2_O_2_	−0.17	0.10	−0.017	−0.22	−0.017
5	RNA: DNA	0.15	−0.17	0.65	0.18	−0.27
6	P450	0.33	0.62	−0.67	−0.35	0.17
7	HSP40	0.13	0.40	−0.22	0.43	0.35

### NMDS results

**Figure 6:**
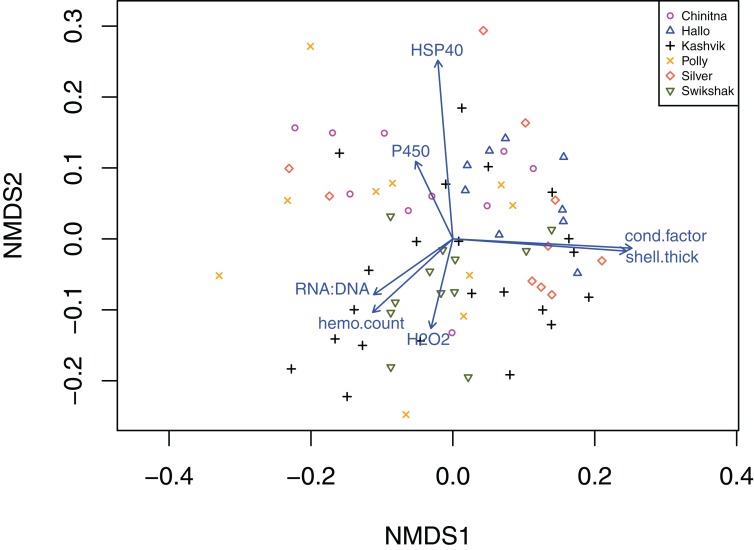
Two-dimensional non-parametric multidimensional scaling plot of the Bray–Curtis dissimilarity from physiological biomarkers. A complete set of seven biomarkers were obtained from 74 razor clams collected in 2015 and 2016 at six sites in Lake Clark and Katmai National Parks and Preserves, Alaska. The vector arrows signify the direction of maximum correlation for each biomarker in the ordination space and are significant at *P* < 0.05. The length of the arrows signifies the strength of the relationship of each metric and the two NMDS metrics, with longer arrows signifying greater strength. Results show that NMDS1 is primarily defined by condition factor, shell thickness, RNA: DNA, and hemocyte count. NMDS 2 is associated with HSP40, P450 and H_2_O_2_. There is no clear clustering by site.

Non-parametric analysis of biomarker data by site yielded results similar to the Pearson correlations analyses (2-dimensional stress = 0.222) (Fig. 6). Results show that NMDS 1 is primarily defined by condition factor, shell thickness, RNA:DNA, and hemocyte count. NMDS 2 is associated with HSP40, P450 and H_2_O_2_. The plot does not clearly show clusters, although there is some separation between two KATM sites (Kashvik and Swikshak) and the rest of the sites on NMDS2.

**Figure 7:**
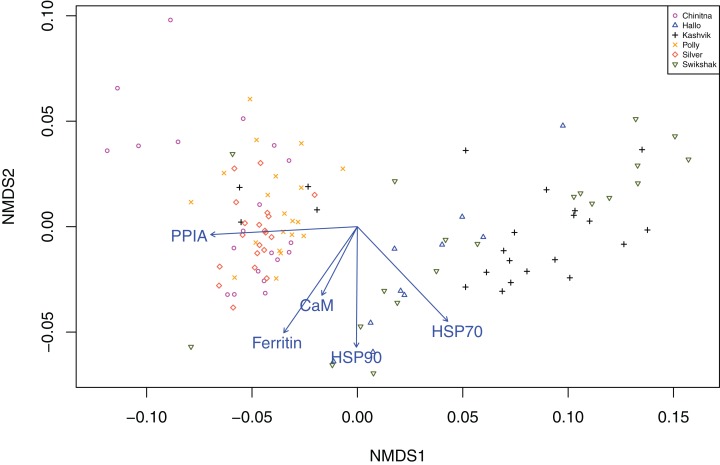
Two-dimensional non-parametric multidimensional scaling plot of the Bray-Curtis dissimilarity from gene transcripts. Gene transcripts were obtained from 112 razor clams collected in 2015 and 2016 at six sites in Lake Clark and Katmai National Parks and Preserves, Alaska. The vector arrows signify the direction of maximum correlation for each gene transcript in the ordination space and are significant *P* < 0.05. The length of the arrow signifies the strength of the relationship of each metric and the two non-metric multidimensional scaling (NMDS) metrics, with longer arrows signifying greater strength. Our results show that NMDS1 is heavily influenced by PPIA and HSP70. NMDS2 is most strongly associated with HSP90. The plot clearly shows two clusters almost entirely divided by park.

Non-parametric multivariate analysis of gene transcription data by site were similar to the Pearson correlation analyses (2-dimensional stress = 0.092) (Fig. 7). Our results show that NMDS 1 is heavily influenced by PPIA and HSP70. NMDS 2 is most strongly associated with HSP 90. The plot clearly shows two clusters almost entirely divided by park. Some individuals from KATM are found in the LACL cluster, but none of the individuals from LACL are found in the KATM cluster.

## Discussion

This was the first study to assess razor clam health as an indicator of nearshore ecosystem health. In most nearshore ecosystem monitoring studies, the physiological responses of bivalves at a study site are compared with bivalves at a non-impacted reference site (Akaishi et al., 2007; Coray, St. Jean & Bard, 2007; Gagné et al., 2007; Halldórsson, Svavarsson & Granmo, 2005; Halldórsson et al., 2008; Shaw et al., 2011). However, when sampling occurred for this study, none of the sites were suspected of being impacted. Rather, our goal was to evaluate a set of biomarker and gene transcription assays which we developed (Objective 1) to assess change in the nearshore ecosystem, which included establishment of reference values for biomarker and gene transcription assays in Pacific razor clams (Objective 2) at sites in LACL and KATM and description of relationships among both assays (Objective 3). Additionally, we wanted to test our hypothesis that Pacific razor clams at all sites were physiologically similar (Objective 4). Although our primary purpose was to evaluate Pacific razor clams as a bioindicator species for long-term monitoring of nearshore marine areas, a further benefit is the acquisition of data that can be used to assess health of individuals and populations of Pacific razor clams, which may support managers who are grappling with fluctuating populations and closures of fisheries (ADF&G, 2010).

Although differences in Pacific razor clams were identified among locations, the variances of physiological and gene transcription values within populations were relatively low when compared to data on bay mussels (*Mytilus trossulus*) collected from the same general locations in the two parks (Counihan et al., 2019). We suspect this may be due in part to lower variability in physical attributes (such as temperature and wave action) of razor clam habitat. Razor clams are infaunal bivalves and predominantly surrounded by interstitial sea water even at low tide. This would preclude them from many of the environmental fluctuations experienced by epifaunal bivalves such as mussels, which have the ability to settle on a variety of intertidal surfaces from bedrock to gravel and are exposed to variations in air temperature, solar radiation and wave action (Helmuth et al., 2010).

### Nutrients

Higher quality and/or quantity of nutrients in the marine intertidal have been associated with increased condition and thicker shells in bivalves (Carmichael, Shriver & Valiela, 2004). The condition factor and shell thickness of Pacific razor clams were higher at LACL than KATM. On average, the condition factor of Pacific razor clams from LACL was 4.7 times higher and shells 1.6 times thicker than Pacific razor clams from KATM. In a concurrent study, LACL mussels had higher condition factors and thicker shells than KATM mussels (Counihan et al., 2019). The condition factor of Pacific razor clams at all KATM sites, except Swikshak in 2015, was below the range observed in Pacific razor clams held in a controlled environment with sufficient nutrients (K. Counihan, 2014, unpublished data). These results suggest that there were differences in nutrient availability and/or quality between parks.

### Pathogen presence

Pathogen exposure generally results in stimulation of the immune system (i.e., increased hemocytes, hydrogen peroxide production, transcription of PPIA and Ferr) (Pipe, 1990; Galloway & Depledge, 2001; Song et al., 2009). The primary immune cells in bivalves are hemocytes and they release reactive oxygen species in response to foreign antigens (Pipe, 1992). Increased immune activity was detected at KATM in 2015, where hemocyte count, hydrogen peroxide production and PPIA and Ferr transcription all were elevated compared to LACL, suggesting an immune response was occurring at KATM. PPIA transcription was consistently elevated at KATM and Pacific razor clams also were in poorer condition compared to LACL; however, causal mechanisms underlying this relationship are not clear. The hemocyte count in Pacific razor clams from Polly Creek in LACL nearly doubled from 2015 to 2016, but there were no concurrent increases in hydrogen peroxide production or PPIA and Ferr transcription. A two-fold or higher increase in hemocyte count has been associated with immune stimulation (Coray, St. Jean & Bard, 2007; Duchemin et al., 2008). Exposure to contaminants has been shown to result in increased hemocyte production without the stimulation of a cell-mediated response (Galloway & Depledge, 2001). However, in other studies, decreased hemocyte production has resulted from exposure to contaminants (Akaishi et al., 2007).

Although we did not attempt to quantify pathogens or algal toxins in this study, both could potentially influence the status of the razor clam populations that we sampled. For example, nuclear inclusion X (NIX) is a disease of serious concern for Pacific razor clams and has decimated populations in Washington state in the past (Elston, 1986). However, NIX is thought to be rare or absent in Alaskan razor clam populations (Meyers & Burton, 2009) and likely was not a factor in this study. Harmful algal blooms, which are increasing at northern latitudes as ocean temperatures warm (Gobler et al., 2017), could also be an issue. Of particular concern are the toxic dinoflagellates in the genus *Alexandrium*, the causative agents of paralytic shellfish poisoning (Vandersea et al., 2018; Tobin et al., 2019). Elevated water temperatures were documented in the Gulf of Alaska in 2015–2016 (Danielson et al., 2019) as well as increased abundance of *Alexandrium* in lower Cook Inlet (Vandersea et al., 2018). Algal toxins are generally recognized as an additional stressor in warming waters (Griffith & Gobler, 2019) and could be a factor in the elevated immune activity detected in razor clams from KATM in 2015 (hemocyte count, H_2_O_2_, and PPIA and Ferr transcription were all elevated). However, further study is needed to ascertain Pacific razor clam responses to algal toxins, and to measure relative toxin exposures in clam populations sampled in different areas.

### Contaminant exposure

Natural oil seeps have been reported throughout Cook Inlet and several are in the vicinity of our sampling sites (Becker & Manen, 1988). A study conducted in 2002 detected PAHs in sediments at multiple coastal sites in southcentral Alaska and the source was attributed to natural seeps (Saupe, Gendron & Dasher, 2005). The cytochrome P450 enzyme catalyzes reactions involving xenobiotics and endogenous compounds (Rewitz et al., 2006; Kingtong & Janvilisri, 2011). Cytochrome P450 activity and HSP40 levels were elevated at Swikshak in KATM and Silver Salmon and Polly Creek in LACL during 2016. HSP40 increases in response to stressors such as contaminants (Wang et al., 2012). The amount of oil seepage can vary over time and could result in variations in P450 activity among sites. However, as we did not measure PAHs at individual sites, we can only speculate about the relationship between P450 and HSP40 levels and PAH presence at Swikshak and other sites.

### Metabolic activity

Metabolic activity is evaluated by quantifying the RNA:DNA ratio, which detects short-term fluctuations in RNA concentration over the course of 1–3 days prior to measurement (Buckley, Caldarone & Ong, 1999; Dahlhoff, 2004). RNA production was 1.5 times higher in KATM Pacific razor clams as compared to LACL Pacific razor clams and was higher at all sites in 2016 compared to 2015. Mussels collected from both parks in 2015 and 2016 also had elevated RNA levels at KATM and during 2016 (Counihan et al., 2019). These results indicate that metabolic activity was heightened at KATM and at both locations during 2016. Protein synthesis may have been induced by multiple factors such as food availability or a stressor (Dahlhoff, 2004). However, shell thickness and condition factor data suggest higher nutrients at LACL; thus increased protein synthesis at KATM is not likely caused by greater food availability. Additionally, all of the sites with higher RNA:DNA ratios in 2016 also had elevated P450 and/or HSP40 levels, suggesting the presence of stressor-stimulated protein production.

### Correlations

Correlations within the biomarker assays, within the gene transcription panel, and among the biomarkers and genes were determined. In the biomarker assays, a positive correlation was found between condition factor and shell thickness. Condition factor indicates the nutritional status of razor clams, and it was not surprising that shell thickness would positively relate to overall condition, as razor clams with more nutrients might be expected to allocate more nutrients to shell formation. In other bivalve species, nutrient deficits have been shown to initiate shell metabolization (Masthanamma, Purushotham & Ramamurthi, 1984).

Given the multiple functions of the genes in our transcript panel, as well as the interconnectedness of genes in general, we expected and found numerous correlations among the genes (Fig. 5). For example, CaM, PPIA and Ferr can all be indicators of pathogen exposure. Although traditionally thought of as a response to thermal stress, HSP70 can also indicate general physiological stress. Thus, as expected, we found positive correlations between CaM and PPIA, CaM and Ferr and PPIA and Ferr. Negative correlations were found between HSP70 and PPIA, as well as HSP70 and Ferr. One potential explanation may be shifts in resource allocation away from one response towards another (Ernande et al., 2004).

As we describe significant differences in physiological and gene transcription results between parks and among sites, it is important to acknowledge the existence of synergistic or antagonistic effects of multiple stressors (Côté, Darling & Brown, 2016; Griffith & Gobler, 2019). Due to the numerous stressors present in any natural environment, it is not possible to correlate every causative influence with an effect on an organism’s physiology. In addition, physiological and genetic pathways are a web of often interconnecting paths; a single gene or physiological response may be initiated by multiple stressors. With multiple assay types (i.e., biomarker and gene transcription), our ability to understand the potential input of both large and fine scale processes improves. For example, our results point, in general, to a higher quantity or quality of nutrients at LACL in comparison with KATM, a higher pathogen response at KATM in comparison with LACL and a higher response to contaminants at Chinitna Bay and Swikshak than at other sites. However, it is also important to note that while we found differences in gene transcription and biomarker assay results between parks and among sites, most or all observations should still be in the range of “normal”.

An objective of our study was to acquire baseline biomarker and gene transcription reference ranges for razor clams. The establishment of baseline or reference ranges in any species is problematic. One option is to use data from captive clinically normal individuals as baseline or reference values. However, captivity comes with its own set of stressors; there is no population free of all external or internal stimuli. Longitudinal data, with the initial timepoint acting as baseline or reference, is an alternative approach. However, the initial measurements, which form the baseline, are simply that: a place from which to make relative comparisons, with the findings in one group compared with other groups. It is important to note in this case that varying levels of transcription within or among groups or individuals may be normal responses to stimuli and an indication of a properly functioning system.

Univariate correlations do not adequately describe the multiple interacting pathways represented by the gene transcripts and biomarkers described above. The vectors generated from our NMDS analysis can be used to evaluate which transcripts and biomarkers give similar information about the sampled population and which, if any, drive group separation in multidimensional space. For example, in our gene transcript analysis, CaM and Ferr seem to describe similar pathways in our populations, while PPIA and HSP90, perpendicular to each other, describe potentially different pathways. Additionally, vectors that are opposite each other on the same axis can be thought of as negative correlations. The size and position of the PPIA vector in relation to the group separation along the NMDS 1 implicate PPIA as a main driver of separation between parks (the differences in PPIA are also evident in the correlation plots, Fig. 5). Although the biomarker analysis failed to separate populations based on park, the vectors indicate that biomarker pairs: condition factor & shell thickness, HSP40 & P450 and RNA:DNA & hemocyte count may describe similar pathways. The gene transcript and biomarker ordinations provided different, but complementary information, suggesting that the combination of gene transcripts and biomarkers in a single ordination model should be a focus of future studies.

Another objective was the comparison of the two methodologies, gene transcription and biomarker assays. Several of the biomarkers and genes were associated with similar physiological functions and we anticipated correlations would arise. Although we did not expect complete agreement or duplication between the methods, we expected the methods to support one another. Ferritin transcription was positively correlated with hemocyte count. This relationship makes biological sense, as an increase in Ferr transcription could indicate a parasite pressure, which could be associated with elevated hemocyte count, an indication of immune stimulus. Negative correlations were found between PPIA and condition factor, as well as PPIA and shell thickness.

A limitation of this study was the low number of genes in the transcript panel. This stems from the fact that relatively little is known about the genetic makeup of invertebrates in comparison with vertebrates (Niu et al., 2013). GenBank^®^, the National Institutes of Health genetic sequence database, an annotated collection of all publicly available DNA sequences, contains only three genetic sequences for the Pacific razor clam, all representing one gene (Clark et al., 2015). The implications of this lack of information are that each “new” target gene must be identified and sequenced using degenerate primers designed from closely-related species (if available) prior to amplification using standard PCR. PCR products are then sequenced and compared with known sequences in GenBank^®^. Herein lies the problem: if GenBank^®^ has a dearth of invertebrate sequences, then comparing a sequence amplified from an equally unexplored invertebrate is likely to yield few matches, if any (Hou et al., 2011). Many of our sequences, when submitted to GenBank^®^, yielded no matches. The results of our considerable efforts were five target genes and two reference genes in which we had complete confidence. Similar difficulties were found in transcriptomic studies of the Chinese razor clam, in which only 9.9% of the 147,669 transcribed sequences had significant matches in GenBank^®^ (Niu et al., 2013).

The limited number of genetic studies on razor clams is incongruent with their value as a commercial species and popularity as a sport fishery. Studies to date have focused on the Chinese razor clam, identifying genes responding to heavy metals (Wang et al., 2016), anthropogenic sound (Peng et al., 2016) and bacterial challenge (Niu et al., 2014, 2016; Peng et al., 2016), and have provided a foundation for the development of protocols for using razor clams as indicators of ecosystem health.

## Conclusions

We hypothesized that biomarker and gene transcription assays focused on Pacific razor clams would provide complementary results, with no significant differences detected among sites considered to be relatively pristine. However, we found differences in physiological assay and gene transcription results between parks and among sites which indicate variation in both large-scale and local environmental conditions. Changing environmental conditions in coastal ecosystems necessitate methods to assess the health of intertidal communities and facilitate management decisions to sustain these resources. Gene transcription assays will be a valuable technique because they provide early evidence of changes in physiologic status. Biomarker assays are an established approach to assess bivalve health. Links between the results of the biomarker and gene transcription assays were observed that support the applicability of both assays in ecosystem monitoring. Further development of gene transcription methods as well as controlled laboratory exposure studies will improve our capacity to monitor for early signs of physiological impacts in razor clams and their ecosystems due to changing environmental conditions.

## Supplemental Information

10.7717/peerj.8761/supp-1 Supplemental Information 1 Medians and ranges for all biomarker variables for all sites and years.

10.7717/peerj.8761/supp-2 Supplemental Information 2 Transcription level medians and ranges for all genes for all sites and years (Note: higher numbers indicate less transcription).

10.7717/peerj.8761/supp-3 Supplemental Information 3 Razor clam biomarker data.

10.7717/peerj.8761/supp-4 Supplemental Information 4 Razor clam gene transcription data.

## Additional Information and Declarations

### Field Study Permissions

The following information was supplied relating to field study approvals (i.e., approving body and any reference numbers): Field experiments were approved by the National Park Service (Permit numbers CF-15-088 and CF-16-089).

### Data Availability

The following information was supplied regarding data availability: The raw measurements are available in the Supplemental Files.

## References

[ref-1] ADF&G (2010). Cook inlet razor clams. http://www.adfg.alaska.gov.

[ref-2] ADF&G (2014). Personal use fishing.

[ref-3] ADF&G (2018). Personal use fishing.

[ref-4] ADF&G (2019). Fishing regulations. http://www.adfg.alaska.gov/index.cfm?adfg=PersonalUsebyAreaSouthcentralcookinletclams.regs.

[ref-5] ADF&G (2020). Razor clam (*Siliqua patula*) species profile. http://www.adfg.alaska.gov/index.cfm?adfg=razorclammain.

[ref-6] Akaishi F, St-Jean S, Bishay F, Clarke J, Rabitto I, De Oliveira Ribeiro C (2007). Immunological responses, histopathological finding and disease resistance of blue mussel (*Mytilus edulis*) exposed to treated and untreated municipal wastewater. Aquatic Toxicology.

[ref-95] Araya MT, Siah A, Mateo D, Markham F, Mckenna P, Johnson G, Berthe FCJ (2008). Selection and evaluation of housekeeping genes for haemocytes of soft-shell clams (*Mya arenaria*) challenged with *Vibrio splendidus*. Journal of Invertebrate Pathology.

[ref-7] Ballachey BE, Bodkin JL, Esler D, Rice SD, Alford JB, Peterson MS, Green CC (2014). Lessons from the 1989 Exxon valdez oil spill: a biological perspective. Impacts of Oil Spill Disasters on Marine Habitats and Fisheries in North America.

[ref-8] Baxter RE (1971). Earthquake effects on clams of Prince William Sound.

[ref-9] Becker P, Manen C (1988). Natural oil seeps in the Alaskan marine environment: final report.

[ref-10] Beyer J, Green NW, Brooks S, Allen IJ, Ruus A, Gomes T, Brate ILN, Schoyen M (2017). Blue mussels (*Mytilus edulis* spp.) as sentinel organisms in coastal pollution monitoring: a review. Marine Environmental Research.

[ref-11] Bishop M, Powers S (2003). Restoration of razor clam (Siliqua patula) populations in southeastern Prince William Sound, Alaska: Integrating science, management and traditional knowledge in the development of a restoration strategy.

[ref-12] Bolognesi C, Cirillo S (2014). Genotoxicity biomarkers in aquatic bioindicators. Current Zoology.

[ref-13] Buckley L, Caldarone E, Ong T-L (1999). RNA-DNA ratio and other nucleic acid-based indicators for growth and condition of marine fishes. Hydrobiologia.

[ref-14] Caldarone EM, Wagner M, Onge-Burns JS, Buckley LJ (2001). Protocol and guide for estimating nucleic acids in larval fish using a fluorescence microplate reader.

[ref-15] Carmichael RH, Shriver AC, Valiela I (2004). Changes in shell and soft tissue growth, tissue composition, and survival of quahogs, *Mercenaria*, and soft shell clams, *Mya arenaria*, in response to eutrophic-driven changes in food supply and habitat. Journal of Experimental Marine Biology and Ecology.

[ref-16] Cellura C, Toubiana M, Roch P (2007). Specific expression of antimicrobial peptide and *HSP70* genes in response to heat-shock and several bacterial challenges in mussels. Fish & Shellfish Immunology.

[ref-17] Chen I-H, Chou L-S, Chou S-J, Wang J-H, Stott J, Blanchard M, Jen I-F, Yang W-C (2015). Selection of suitable reference genes for normalization of quantitative RT-PCR in peripheral blood samples of bottlenose dolphins (*Tursiops truncatus*). Scientific Reports.

[ref-18] Chen Z-F, Wang H, Matsumura K, Qian P-Y (2012). Expression of calmodulin and myosin light chain kinase during larval settlement of the barnacle *Balanus amphitrite*. PLOS ONE.

[ref-19] Choi YK, Jo PG, Choi CY (2008). Cadmium affects the expression of heat shock protein 90 and metallothionein mRNA in the Pacific oyster, *Crassostrea gigas*. Comparative Biochemistry and Physiology—Part C: Toxicology & Pharmacology.

[ref-20] Choi CY, Min BH, Kim NN, Cho SH, Chang YJ (2006). Expression of HSP90, HSP70 mRNA and change of plasma cortisol and glucose during water temperature rising in freshwater adapted black porgy, *Acanthopagrus schlegeli*. Journal of Aquaculture.

[ref-21] Clark K, Karsch-Mizrachi I, Lipman DJ, Ostell J, Sayers EW (2015). GenBank. Nucleic Acids Research.

[ref-22] Coray C, St. Jean S, Bard S (2007). Immunomodulation in blue mussels (*Mytilus edulis* and *Mytilus trossulus*) exposed to municipal effluent in Eastern Canada. Water Quality Research Journal.

[ref-23] Counihan KL (2018). The physiological effects of oil, dispersant and dispersed oil on the bay mussel, Mytilus trossulus, in Arctic/Subarctic conditions. Aquatic Toxicology.

[ref-24] Counihan K, Bowen L, Ballachey B, Coletti H, Hollmen T, Pister B (2019). Physiological and gene transcription assays in combination: a new paradigm for marine intertidal assessment. PeerJ.

[ref-25] Cruz-Rodriguez LA, Chu F-LE (2002). Heat-shock protein (HSP70) response in the eastern oyster, Crassostrea virginica, exposed to PAHs absorbed to suspended artificial clay particles and to suspended field contaminated sediments. Aquatic Toxicology.

[ref-26] Côté IM, Darling ES, Brown CJ (2016). Interactions among ecosystem stressors and their importance in conservation. Proceedings of the Royal Society B: Biological Sciences.

[ref-27] Dahlhoff EP (2004). Biochemical indicators of stress and metabolism: applications for marine. Annual Review of Physiology.

[ref-28] Danielson SL, Hennon TD, Monson DH, Suryan RM, Campbell RW, Baird SJ, Holderied K, Weingartner TJ, Suryan MR, Lindeberg MR, Aderhold DR (2019). Chapter 1: a study of marine temperature variations in the northern Gulf of Alaska across years of marine heatwaves and cold spells. Gulf Watch Alaska Long-Term Monitoring Program Draft Synthesis Report: The Pacific Marine Heatwave: Monitoring During a Major Perturbation in the Gulf of Alaska.

[ref-29] Dean T, Bodkin J (2006). Sampling protocol for the nearshore restoration and ecosystem monitoring (NREM) program.

[ref-30] Dean TA, Bodkin JL, Coletti HA (2014). Protocol Narrative for Nearshore Marine Ecosystem Monitoring in the Gulf of Alaska, version 1.1.

[ref-31] Di Lorenzo E, Mantua N (2016). Multi-year persistence of the 2014/15 North Pacific marine heatwave. Nature Climate Change.

[ref-32] Duchemin M, Auffret M, Wessel N, Fortier M, Morin Y, Pellerin J, Fournier M (2008). Multiple experimental approaches of immunotoxic effects of mercury chloride in the blue mussel, *Mytilus edulis*, through in vivo, in tubo and in vitro exposures. Environmental Pollution.

[ref-33] Elston RA (1986). An intranuclear pathogen [nuclear inclusion X (NIX)] associated with massive mortalities of the Pacific razor clam, *Siliqua patula*. Journal of Invertebrate Pathology.

[ref-34] Ernande B, Boudry P, Clobert J, Haure J (2004). Plasticity in resource allocation based life history traits in the Pacific oyster, Crassostrea gigas. I. Spatial variation in food abundance. Journal of Evolutionary Biology.

[ref-35] Esler D, Ballachey BE, Matkin C, Cushing D, Kaler R, Bodkin J, Monson D, Esslinger G, Kloecker K (2017). Timelines and mechanisms of wildlife population recovery following the Exxon Valdez oil spill. Deep Sea Research Part II.

[ref-36] Evans TG, Hofmann GE (2012). Defining the limits of physiological plasticity: how gene expression can assess and predict the consequences of ocean change. Philosophical Transactions of the Royal Society B: Biological Sciences.

[ref-37] Farr S, Dunn RT (1999). Concise review: gene expression applied to toxicology. Toxicological Sciences.

[ref-38] Foster NR (1991). Intertidal bivalves: a guide to the common marine bivalves of Alaska.

[ref-39] Gagné F, Blaise C, André C, Gagnon C, Salazar M (2007). Neuroendocrine disruption and health effects in *Elliptio complanata* mussels exposed to aeration lagoons for wastewater treatment. Chemosphere.

[ref-40] Gagné F, Blaise C, Aoyama I, Luo R, Gagnon C, Couillard Y, Campbell P, Salazar M (2002). Biomarker study of a municipal effluent dispersion plume in two species of freshwater mussels. Environmental Toxicology.

[ref-41] Gagné F, Burgeot T, Hellou J, St-Jean S, Farcy E, Blaise C (2008). Spatial variations in biomarkers of *Mytilus edulis* mussels at four polluted regions spanning the Northern Hemisphere. Environmental Research.

[ref-42] Galloway TS, Depledge MH (2001). Immunotoxicity in invertebrates: measurement and ecotoxicological relevance. Ecotoxicology.

[ref-43] Gao Q, Song L, Ni D, Wu L, Zhang H, Chang Y (2007). cDNA cloning and mRNA expression of heat shock protein 90 gene in the haemocytes of Zhikong scallop *Chlamys farreri*. Comparative Biochemistry and Physiology Part B: Biochemistry and Molecular Biology.

[ref-44] Gaylord B, Hill T, Sanford E, Lenz E, Jacobs L, Sato K, Russell A, Hettinger A (2011). Functional impacts of ocean acidification in an ecologically critical foundation species. Journal of Experimental Biology.

[ref-45] Gobler CJ, Doherty OM, Hattenrath-Lehmann TK, Griffith AW, Kang Y, Litaker RW (2017). Ocean warming since 1982 has expanded the niche of toxic algal blooms in the North Atlantic and North Pacific oceans. Proceedings of the National Academy of Sciences of the United States of America.

[ref-46] Griffith AW, Gobler CJ (2019). Harmful algal blooms: a climate change co-stressor in marine and freshwater ecosystems. Harmful Algae.

[ref-47] Halldórsson HP, De Pirro M, Romano C, Svavarsson J, Sarà G (2008). Immediate biomarker responses to benzo[a]pyrene in polluted and unpolluted population of the blue mussel (*Mytilus edulis* L.) at high-latitudes. Environment International.

[ref-48] Halldórsson HP, Svavarsson J, Granmo Å (2005). The effect of pollution on scope for growth of the mussel (*Mytilus edulis* L.) in Iceland. Marine Environmental Research.

[ref-50] Helmuth B, Broitman BR, Yamane L, Gilman S, Mach K, Mislan KA, Denny MW (2010). Organismal climatology: analyzing environmental variability at scales relevant to physiological stress. Journal of Experimental Biology.

[ref-51] Hou R, Bao Z, Wang S, Su H, Li Y, Du H, Hu J, Wang S, Hu X (2011). Transcriptome sequencing and de novo analysis for Yesso scallop (*Patinopecten yessoensis*) using 454 GS FLX. PLOS ONE.

[ref-52] Kaiser J, Konigsberg D (1977). Razor clam (Siliqua patula) distribution and population assessment study.

[ref-53] Kingtong S, Janvilisri T, McGevin L (2011). Molecular determinants in mussels as biomarkers for environmental stress. Anatomy, Habitat and Environmental Impact.

[ref-54] Leite RB, Milan M, Coppe A, Bortoluzzi S, Dos Anjos A, Reinhardt R, Saavedra C, Patarnello T, Cancela ML, Bargelloni L (2013). mRNA-Seq and microarray development for the Grooved carpet shell clam, *Ruditapes decussatus*: a functional approach to unravel host-parasite interaction. BMC Genomics.

[ref-55] Lesser M, Bailey M, Merselis D, Morrison J (2010). Physiological response of the blue mussel *Mytilus edulis* to differences in food and temperature in the Gulf of Maine. Comparative Biochemistry and Physiology Part A: Molecular & Integrative Physiology.

[ref-56] Li C, Li H, Su X, Li T (2011). Identification and characterization of a clam ferritin from *Sinonovacula constricta*. Fish & Shellfish Immunology.

[ref-57] Livingstone DR, Chipman JK, Lowe DM, Minier C, Pipe RK (2000). Development of biomarkers to detect the effects of organic pollution on aquatic invertebrates: recent molecular, genotoxic, cellular and immunological studies on the common mussel (*Mytilus edulis* L.) and other mytilids. International Journal of Environment and Pollution.

[ref-58] Masthanamma P, Purushotham K, Ramamurthi R (1984). Metabolism of Lamellidens marginalis (Lamarck) in relation to starvation stress. I. Mortality, body weight, and oxygen consumption. Indian Journal of Comparative Animal Physiology.

[ref-96] Mateo DR, Greenwood SJ, Araya MT, Berthe FCJ, Johnson GR, Siah A (2010). Differential gene expression of y-actin, Toll-like receptor 2 (TLR-2) and interleukin-1 receptor-associated kinase 4 (IRAK-4) in Mya arenaria haemocytes induced by *in vivo* infections with two *Vibrio splendidus* strains. Developmental and Comparative Immunology.

[ref-59] McLoughlin K, Turteltaub K, Bankaitis-Davis D, Gerren R, Siconolfi L, Storm K, Cheronis J, Trollinger D, Macejak D, Tryon V, Bevilacqua M (2006). Limited dynamic range of immune response gene expression observed in healthy blood donors using RT-PCR. Molecular Medicine.

[ref-60] Mensah-Osman E, Thomas D, Tabb M, Larios J, Hughes D, Giordano T, Lizyness M, Rae JM, Blumberg B, Hollenberg PF, Baker LH (2007). Expression levels and activation of a PXR variant are directly related to drug resistance in osteosarcoma cell lines. Cancer.

[ref-61] Meyers T, Burton T (2009). Diseases of wild and cultured shellfish in Alaska.

[ref-62] Miller LP, Dowd WW (2019). Repeatable patterns of small-scale spatial variation in intertidal mussel beds and their implications for responses to climate change. Comparative Biochemistry and Physiology Part A: Molecular & Integrative Physiology.

[ref-63] Miller KM, Li S, Kaukinen KH, Ginther N, Hammill E, Curtis JMR, Patterson DA, Sierocinski T, Donnison L, Pavlidis P, Hinch SG, Hruska KA, Cooke SJ, English KK, Farrell AP (2011). Genomic signatures predict migration and spawning failure in wild Canadian salmon. Science.

[ref-97] Moreira R, Balseiro P, Romero A, Dios S, Posada D, Novoa B, Figueras A (2012). Gene expression analysis of clams *Ruditapes philippinarum* and *Ruditapes decussatus* following bacterial infection yields molecular insights into pathogen resistance and immunity. Developmental & Comparative Immunology.

[ref-64] Mos L, Cooper GA, Serben K, Cameron M, Koop BF (2008). Effects of diesel on survival, growth, and gene expression in rainbow trout (*Oncorhynchus mykiss*) fry. Environmental Science and Technology.

[ref-65] Niu D, Wang L, Sun F, Liu Z, Li J (2013). Development of molecular resources for an intertidal clam, *Sinonovacula constricta*, using 454 transcriptome sequencing. PLOS ONE.

[ref-66] Niu D, Wang F, Zhao H, Wang Z, Xie S, Li J (2016). Identification, expression, and innate immune responses of two insulin-like peptide genes in the razor clam *Sinonovacula constricta*. Fish & Shellfish Immunology.

[ref-67] Niu D, Xie S, Bai Z, Wang L, Jin K, Li J (2014). Identification, expression, and responses to bacterial challenge of the cathepsin C gene from the razor clam *Sinonovacula constricta*. Developmental & Comparative Immunology.

[ref-68] National Marine Fisheries Service (2016). Annual commercial landings statistics. http://www.st.nmfs.noaa.gov/st1/commercial/landings/annual_landings.html.

[ref-69] Peng C, Zhao X, Liu S, Shi W, Han Y, Guo C, Jiang J, Wan H, Shen T, Liu G (2016). Effects of anthropogenic sound on digging behavior, metabolism, Ca2+/Mg2+ ATPase activity, and metabolism-related gene expression of the bivalve *Sinonovacula constricta*. Science Reports.

[ref-70] Peng C, Zhao X, Liu S, Shi W, Han Y, Guo C, Peng X, Chai X, Liu G (2017). Ocean acidification alters the burrowing behaviour, Ca2+/Mg2+-ATPase activity, metabolism, and gene expression of a bivalve species, *Sinonovacula constricta*. Marine Ecology Progress Series.

[ref-71] Perrigault M, Tanguy A, Allam B (2009). Identification and expression of differentially expressed genes in the hard clam, *Mercenaria mercenaria*, in response to quahog parasite unknown (QPX). BMC Genomics.

[ref-72] Pipe RK (1990). Hydrolytic enzymes associated with the granular haemocytes of the marine mussel *Mytilus edulis*. Histochemical Journal.

[ref-73] Pipe RK (1992). Generation of reactive oxygen metabolites by the haemocytes of the mussel *Mytilus edulis*. Developmental & Comparative Immunology.

[ref-74] Pipe RK, Coles JA (1995). Environmental contaminants influencing immune function in marine bivalve molluscs. Fish & Shellfish Immunology.

[ref-75] Poynton HC, Vulpe CD (2009). Ecotoxicogenomics: emerging technologies for emerging contaminants. Journal of the American Water Resources Association.

[ref-76] Qi D, Chen L, Chen B, Gao Z, Zhong W, Feely RA, Anderson LG, Sun H, Chen J, Chen M, Zhan L, Zhang Y, Cai WJ (2017). Increase in acidifying water in the western Arctic Ocean. Nature Climate Change.

[ref-77] R Development Core Team (2012). R: a language and environment for statistical computing.

[ref-78] R Development Core Team (2018). R: a language and environment for statistical computing.

[ref-79] Rewitz KF, Styrishave B, Lobner-Olesen A, Andersen O (2006). Marine invertebrate cytochrome P450: emerging insights from vertebrate and insect analogies. Comparative Biochemistry and Physiology Part C: Toxicology & Pharmacology.

[ref-80] Saeedi H, Costello MJ (2019). The biology, ecology, and societal importance of razor clams. Reference Module in Earth Systems and Environmental Sciences.

[ref-81] Saupe S, Gendron J, Dasher D (2005). The condition of southcentral Alaska coastal bays and estuaries.

[ref-82] Sforzini S, Oliveri C, Orru A, Chessa G, Pacchioni B, Millino C, Jha AN, Viarengo A, Banni M (2018). Application of a new targeted low density microarray and conventional biomarkers to evaluate the health status of marine mussels: a field study in Sardinian coast, Italy. Science of the Total Environment.

[ref-83] Shaw JP, Dondero F, Moore MN, Negri A, Dagnino A, Readman JW, Lowe DR, Frickers PE, Beesley A, Thain JE, Viarengo A (2011). Integration of biochemical, histochemical and toxicogenomic indices for the assessment of health status of mussels from the Tamar Estuary, UK. Marine Environmental Research.

[ref-84] Smith T, Partridge S (2004). Dynamics of intertidal foraging by coastal brown bears in southwestern Alaska. Journal of Wildlife Management.

[ref-85] Snell TW, Brogdon SE, Morgan MB (2003). Gene expression profiling in ecotoxicology. Ecotoxicology.

[ref-86] Song X, Wang L, Song L, Zhao J, Zhang H, Zheng P, Qiu L, Liu X, Wu L (2009). A cyclophilin A inducible expressed in gonad of zhikong scallop *Chlamys farreri*. Molecular Biology Reports.

[ref-87] Szarzi NJ, Kerkvliet CM, Failor BJ, Booz MD (2010). Recreational fisheries in the Lower Cook Inlet Management Area, 2008-2010, with updates for 2007.

[ref-88] Tobin ED, Wallace CI, Crumpton C, Johnson G, Eckert GI (2019). Environmental drivers of paralytic shellfish toxin producing *Alexandrium catenella* blooms in a fjord system of northern Southeast Alaska. Harmful Algae.

[ref-89] Vandersea MW, Kibler SR, Tester PA, Holderied K, Hondolero DE, Powell K, Baird S, Doroff A, Dugan D, Litaker RW (2018). Environmental factors influencing the distribution and abundance of Alexandrium catenella in Kachemak bay and lower cook inlet, Alaska. Harmful Algae.

[ref-90] Versteegh L, Hansson T (2012). Assessment of shell thickness in Mytilus edulis as a biomarker for establishment of its health and thiamine status. http://www.raysforexcellence.se/.

[ref-91] Wang R, Li C, Stoeckel J, Moyer G, Liu Z, Peatman E (2012). Rapid development of molecular resources for a freshwater mussel, *Villosa lienosa* (Bivalvia: Unionidae), using an RNA-seq-based approach. Freshwater Science.

[ref-92] Wang Z, Shao Y, Li C, Zhang W, Duan X, Zhao X, Qiu Q, Jin C (2016). RNA-seq analysis revealed ROS-mediated related genes involved in cadmium detoxification in the razor clam *Sinonovacula constricta*. Fish & Shellfish Immunology.

[ref-93] Wang M, Zhang D, Wang L, Gai Y, Zhou Z, Zhang H, Song L (2013). The molecular characterization of a cyclophilin A from Chinese mitten crab *Eriocheir sinensis* and the antifungal activity of its recombinant protein. Electronic Journal of Biotechnology.

[ref-94] Wu J, Basha MR, Brock B, Cox DP, Cardozo-Pelaez F, McPherson CA, Harry J, Rice DC, Maloney B, Chen D, Lahiri DK, Zawai NH (2008). Alzheimer’s disease (AD)-like pathology in aged monkeys after infantile exposure to environmental metal lead (Pb): evidence for a developmental origin and environmental link for AD. Journal of Neuroscience.

