# Supplementary material for: Monitoring nearshore ecosystem health using Pacific razor clams (Siliqua patula) as an indicator species
Source: PeerJ. 2020 Mar 5;8:e8761. doi: 10.7717/peerj.8761 (PMC7060925; doi:10.7717/peerj.8761)
Supplement: Supplemental Information 1 [file peerj-08-8761-s001.docx]

Supplemental Table S1: Medians and ranges for all biomarker variables for all sites and years

|  |  |  |  | Condition Factor | | Shell Thickness | | Hemocyte Count | | Hydrogen Peroxide | |
| --- | --- | --- | --- | --- | --- | --- | --- | --- | --- | --- | --- |
| Park | Site | Year | N | Median | Range | Median | Range | Median | Range | Median | Range |
| Katmai | Hallo Bay | 2015 | NA | NA | NA | NA | NA | NA | NA | NA | NA |
|  |  | 2016 | 12 | 0.07 | 0.05-0.65 | 0.20 | 0.13-0.40 | 6.42 | 1.17-40.83 | 0.04 | 0.02-0.08 |
|  | Kashvik Bay | 2015 | 10 | 0.25 | 0.12-0.29 | 0.34 | 0.17-0.48 | 29.79 | 6.33-58.33 | 0.05 | 0.03-0.12 |
|  |  | 2016 | 12 | 0.14 | 0.08-0.66 | 0.28 | 0.17-0.78 | 10.33 | 1.83-33.00 | 0.04 | 0.01-0.06 |
|  | Swikshak Bay | 2015 | 12 | 0.30 | 0.23-0.37 | 0.35 | 0.18-0.48 | 10.42 | 2.50-88.83 | 0.09 | 0.04-0.12 |
|  |  | 2016 | 10 | 0.18 | 0.05-0.64 | 0.18 | 0.11-0.68 | 8.67 | 0.67-55.83 | 0.07 | 0.04-0.11 |
| Lake Clark | Polly Creek | 2015 | 10 | NA | NA | NA | NA | 4.42 | 2.00-29.50 | 0.03 | 0.02-0.05 |
|  |  | 2016 | 10 | 0.79 | 0.69-1.35 | 0.45 | 0.28-0.79 | 9.33 | 3.67-24.50 | 0.03 | 0.02-0.05 |
|  | Silver Salmon | 2015 | 10 | 0.92 | 0.68-1.14 | 0.51 | 0.41-0.64 | 6.67 | 2.00-163.50 | 0.05 | 0.03-0.11 |
|  |  | 2016 | 10 | 0.66 | 0.40-1.22 | 0.39 | 0.25-0.75 | 9.09 | 4.67-10.20 | 0.07 | 0.04-0.08 |
|  | Chinitna Bay | 2015 | 10 | NA | NA | NA | NA | 20.58 | 10.00-43.67 | 0.06 | 0.02-0.08 |
|  |  | 2016 | 10 | 1.07 | 0.66-1.91 | 0.34 | 0.26-1.90 | 17.58 | 6.33-107.17 | 0.06 | 0.04-0.08 |
|  |  | | | | | | | | | | |
|  |  |  |  | RNA:DNA | | P450 | | HSP40 | |  |  |
| Park | Site | Year | N | Median | Range | Median | Range | Median | Range |  |  |
| Katmai | Hallo Bay | 2015 | NA | NA | NA | NA | NA | NA | NA |  |  |
|  |  | 2016 | 12 | 321.93 | 14.23-448.95 | 49.33 | 17.32-67.75 | 2.36 | 0.00-4.15 |  |  |
|  | Kashvik Bay | 2015 | 10 | 36.87 | 24.10-265.19 | 38.13 | 17.91-64.51 | 0.22 | 0.00-1.65 |  |  |
|  |  | 2016 | 12 | 59.59 | 19.09-326.40 | 35.47 | 13.11-55.51 | 2.77 | 0.99-7.87 |  |  |
|  | Swikshak Bay | 2015 | 12 | 39.74 | 19.51-92.98 | 38.73 | 13.29-78.37 | 0.79 | 0.18-2.73 |  |  |
|  |  | 2016 | 10 | 139.52 | 50.66-454.10 | 70.99 | 24.25-146.37 | 2.41 | 0.32-6.36 |  |  |
| Lake Clark | Polly Creek | 2015 | 10 | 35.90 | 13.80-99.52 | 52.82 | 25.82-96.53 | 0.99 | 0.01-2.56 |  |  |
|  |  | 2016 | 10 | 87.32 | 20.02-152.66 | 65.70 | 26.21-136.81 | 2.41 | 0.39-4.64 |  |  |
|  | Silver Salmon | 2015 | 10 | 10.78 | 4.91-32.99 | 38.94 | 24.17-95.82 | 0.99 | 0.21-1.67 |  |  |
|  |  | 2016 | 10 | 156.46 | 28.73-260.62 | 59.32 | 17.15-107.87 | 1.80 | 0.59-17.79 |  |  |
|  | Chinitna Bay | 2015 | 10 | 17.53 | 0.63-69.00 | 50.85 | 36.58-78.11 | 1.09 | 0.14-1.71 |  |  |
|  |  | 2016 | 10 | 147.84 | 82.28-176.49 | 35.01 | 16.73-132.95 | 1.81 | 0.22-7.14 |  |  |
