# Supplementary material for: Monitoring nearshore ecosystem health using Pacific razor clams (Siliqua patula) as an indicator species
Source: PeerJ. 2020 Mar 5;8:e8761. doi: 10.7717/peerj.8761 (PMC7060925; doi:10.7717/peerj.8761)
Supplement: Supplemental Information 2 [file peerj-08-8761-s002.docx]

Supplemental Table S2: Transcription level medians and ranges for all genes for all sites and years (Note: higher numbers indicate less transcription).

|  |  |  |  | Calmodulin | | Ferritin | | HSP70 | | HSP90 | |
| --- | --- | --- | --- | --- | --- | --- | --- | --- | --- | --- | --- |
| Park | Site | Year | N | Median | Range | Median | Range | Median | Range | Median | Range |
| Katmai | Hallo Bay | 2015 | NA | NA | NA | NA | NA | NA | NA | NA | NA |
|  |  | 2016 | 10 | 8.60 | 7.14-9.88 | 1.51 | -0.07-3.02 | 5.31 | 4.80-6.91 | 5.69 | 3.14-6.22 |
|  | Kashvik Bay | 2015 | 10 | 7.60 | 7.12-9.47 | -0.37 | -1.56-1.26 | 6.88 | 5.31-7.87 | 5.40 | 4.22-6.46 |
|  |  | 2016 | 12 | 7.30 | 6.28-8.84 | 0.61 | -0.55-1.36 | 6.35 | 4.43-7.68 | 5.89 | 4.50-6.26 |
|  | Swikshak Bay | 2015 | 10 | 7.51 | 6.91-8.13 | -0.52 | -1.35-0.69 | 7.19 | 5.36-8.03 | 4.43 | 2.96-5.72 |
|  |  | 2016 | 10 | 8.35 | 6.88-10.39 | 1.87 | 1.12-3.78 | 5.29 | 4.66-5.79 | 5.58 | 3.11-6.41 |
| Lake Clark | Polly Creek | 2015 | 10 | 7.86 | 6.72-8.24 | 1.18 | 0.43-1.98 | 4.42 | 2.71-6.47 | 5.97 | 4.66-6.46 |
|  |  | 2016 | 10 | 8.00 | 7.18-9.09 | 1.17 | 0.20-2.51 | 5.46 | 4.01-5.88 | 5.33 | 4.50-6.02 |
|  | Silver Salmon | 2015 | 10 | 8.53 | 7.54-8.72 | 1.38 | 0.82-1.77 | 4.99 | 3.74-5.63 | 5.16 | 4.22-5.99 |
|  |  | 2016 | 10 | 8.44 | 7.61-9.62 | 1.58 | 1.14-1.90 | 5.37 | 4.81-6.02 | 5.75 | 4.67-6.62 |
|  | Chinitna Bay | 2015 | 10 | 8.60 | 7.66-10.25 | 0.94 | 0.37-2.35 | 3.76 | 1.52-5.28 | 3.11 | 2.05-5.10 |
|  |  | 2016 | 10 | 8.67 | 7.60-9.40 | 1.43 | 0.61-1.98 | 5.69 | 4.97-6.34 | 6.37 | 5.35-6.69 |
|  |  | | | | | | | | | | |
|  |  |  |  | PPIA | |  |  |  |  |  |  |
| Park | Site | Year | N | Median | Range |  |  |  |  |  |  |
| Katmai | Hallo Bay | 2015 | NA | NA | NA |  |  |  |  |  |  |
|  |  | 2016 | 10 | 3.65 | 2.02-5.21 |  |  |  |  |  |  |
|  | Kashvik Bay | 2015 | 10 | 1.92 | 0.50-7.43 |  |  |  |  |  |  |
|  |  | 2016 | 12 | 2.49 | 1.25-8.75 |  |  |  |  |  |  |
|  | Swikshak Bay | 2015 | 10 | 0.80 | 0.05-3.19 |  |  |  |  |  |  |
|  |  | 2016 | 10 | 4.32 | 2.25-10.36 |  |  |  |  |  |  |
| Lake Clark | Polly Creek | 2015 | 10 | 8.13 | 7.18-8.50 |  |  |  |  |  |  |
|  |  | 2016 | 10 | 8.03 | 6.98-9.23 |  |  |  |  |  |  |
|  | Silver Salmon | 2015 | 10 | 8.44 | 7.63-9.09 |  |  |  |  |  |  |
|  |  | 2016 | 10 | 8.80 | 7.55-9.61 |  |  |  |  |  |  |
|  | Chinitna Bay | 2015 | 10 | 8.20 | 7.49-9.30 |  |  |  |  |  |  |
|  |  | 2016 | 10 | 8.58 | 8.17-9.43 |  |  |  |  |  |  |
